# Wood Warping Composite by 3D Printing

**DOI:** 10.3390/polym14040733

**Published:** 2022-02-14

**Authors:** Doron Kam, Ido Levin, Yinnon Kutner, Omri Lanciano, Eran Sharon, Oded Shoseyov, Shlomo Magdassi

**Affiliations:** 1 Institute of Chemistry, The Hebrew University of Jerusalem, Jerusalem 9190401, Israel; doron.kam@mail.huji.ac.il (D.K.); yinnon.kutner@gmail.com (Y.K.); omrilanciano@gmail.com (O.L.); 2 Plant Sciences and Genetics in Agriculture, the Hebrew University of Jerusalem, Rehovot 7610001, Israel; 3 Racah Institute of Physics, the Hebrew University, Jerusalem 9190401, Israel; ido.levin@mail.huji.ac.il; 4 Alpha Program, Future Scientist Center, Jerusalem 9190401, Israel

**Keywords:** 3D printing, nanocellulose, shape programming, wood

## Abstract

Wood warping is a phenomenon known as a deformation in wood that occurs when changes in moisture content cause an unevenly volumetric change due to fiber orientation. Here we present an investigation of wood warped objects that were fabricated by 3D printing. Similar to natural wood warping, water evaporation causes volume decrease of the printed object, but in contrast, the printing pathway pattern and flow rate dictate the direction of the alignment and its intensity, all of which can be predesigned and affect the resulting structure after drying. The fabrication of the objects was performed by an extrusion-based 3D printing technique that enables the deposition of water-based inks into 3D objects. The printing ink was composed of 100% wood-based materials, wood flour, and plant-extracted natural binders cellulose nanocrystals, and xyloglucan, without the need for any additional synthetic resins. Two archetypal structures were printed: cylindrical structure and helices. In the former, we identified a new length scale that gauges the effect of gravity on the shape. In the latter, the structure exhibited a shape transition analogous to the opening of a seedpod, quantitatively reproducing theoretical predictions. Together, by carefully tuning the flow rate and printing pathway, the morphology of the fully dried wooden objects can be controlled. Hence, it is possible to design the printing of wet objects that will form different final 3D structures.

## 1. Introduction

Wood is processed by subtractive manufacturing, in which a tree trunk is first cut down, and then sawn into smaller pieces. Traditionally, these pieces form the building blocks from which wood products can be built, or in a more modern approach, these pieces are chemically and mechanically processed with additives to produce wood plate materials (e.g., MDF or plywood). These processes influence the way we design our products, providing guidelines to optimize costs and enhance dimensional stability. For example, layered wooden plates are both cheap and mechanically resilient. Therefore, whenever a wooden structure contains a curved, thin part, it is formed by bending a flat plate. This limits the part to bend only in one direction, and objects that must have a double curvature, i.e., the seat of a chair, cannot be built from a plate, thus increasing their complexity and price [1]. This is well known by carpenters and cabinetmakers.

Here, we invert this subtractive manufacturing into additive manufacturing by 3D printing natural plant cell-wall binders and wood particles [2]. Our fabrication approach enables creating or reconstructing composites without “paying” in labor for their complexity. This enables us to revolutionize the way we design and fabricate wooden structures. By our approach, we 3D print a liquid dispersion wooden ink that later dries out and solidifies. Traditionally, this can be problematic as it leads to a distortion of the shape after the fabrication of the 3D object. Moreover, in many cases, a non-uniform shrinkage can lead to material failure (i.e., in drying mud, internal stresses caused by humidity gradients lead to fracture).

**Figure 1:**
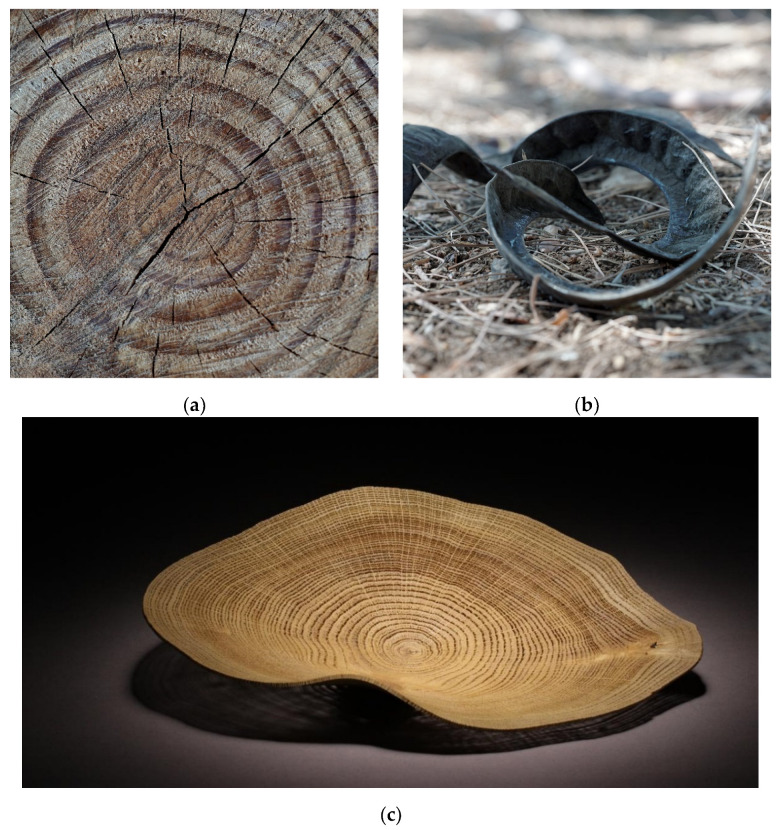
Examples of shrinking-induced shape changes in drying natural materials. (**a**) Crack propagation in *Pinus halepensis* tree trunk due to volume decrease by water evaporation in a cylindrical geometry constrain. (**b**) *Delonix regia* seed pod twists into a chiral shape. (**c**) A drying thin slice of an oak tree trunk buckles out-of-plane releasing the internal stresses instead of breaking (credit: Pascal Oudet [7]).

For instance, this is often the case when a tree is cut down. The loss of water causes shrinkage along the three axes as moisture content changes [3]. Wood internal structure consists mainly of longitudinal tracheid cells and about 5% radially oriented ray cells [4]. Therefore, shrinkage in volume imposes an axisymmetric shrinkage that is different in the radial and longitudinal direction and frequently leads to the development of cracks (Figure 1a) [5]. Such uneven shrinkage is a phenomenon known as wood warping. This phenomenon, as beautiful as it is, does not only damage products made of wood, such as in house constructions and furniture, but harms engineering tolerances [6].

Nevertheless, the very same mechanism is exploited by members of the plant kingdom to change their shape. Plants have no muscles or skeletons and rely on differential growth to achieve shape changes [8,9]. Moreover, when a plant dries and shriveled due to water evaporation, which reduces its volume, cell wall constraints dictate the features of the resulting deformation [10,11]. These constraints may take a functional role of movement, for spreading seeds or digging in the sand for germination (Figure 1b).

Recently, this phenomenon was adopted to fabricate biomimetic responsive materials that undergo uneven shrinkage as a means to induce shape changes in thin sheets, which favor buckling out-of-plane, rather than breaking [12]. Consequently, a thin tree trunk slice may buckle out-of-plane instead of breaking, as shown in Figure 1c. An important class of smart materials that undergoes an anisotropic shrinkage includes nematic elastomers [13], 3D printed thermoplastics [14], hydrogels [15], and channeled inflatables [16]. Extrusion-based 3D printing, as investigated in the current research, also belongs to this category, for which previous reports have identified two main factors that govern the programmed geometry: the amount of shrinkage anisotropy and its local orientation [17]. The first corresponds to the amount of the induced alignment and the latter to the printing direction. Therefore, controlling the alignment direction is straightforward by pre-programming the orientation by various techniques. However, direct control of the alignment itself is not simple and is possible only in a few experimental systems [18].

In this report, we utilized a 3D printing approach to control the alignment direction and the printing flow rate to control the shrinkage anisotropy of wood objects. The printing speed was selected as a control parameter since it was assumed that the alignment of the materials in the ink is dependent on flow rate. Aiming for a wood-like material, we used cellulose-based ink composed of wood-waste microparticles, named wood flour (WF), and the plant-extracted natural binders, cellulose nanocrystals (CNCs) and xyloglucan (XG). Like in natural wood, we also use water as the main component in building structures that, through water evaporation, results in a 3D warped object. The controlled change in structure to water evaporation opens the way towards the 4D printing of fully natural wood objects.

## 2. Materials and Methods

Cellulose nanocrystal (CNC) freeze-dried powder was obtained from Celluforce Inc., Montreal, Canada (freeze-dry, LOT #2015-009). Wood flour (WF) from hardwood (particle size distribution of 92% < 75 µm and 2% > 150 µm) was obtained as a gift from LA.SO.LE. EST S.P.A, Percoto, Italy (MOD-EASY FIBER-75). Xyloglucan (XG) from tamarind seed was supplied by Megazyme Inc., Bray, Ireland (Lot #150901).

2 wt% XG suspensions were prepared by vigorously mixing XG with distilled water (DW) in a glass vial for 1 h, at 80 °C, until a clear suspension was achieved. CNC was suspended in DW (7.5 wt%) and sonicated. Wood ink was prepared by mixing WF:CNC:XG (2.9:1:0.01 wt) for 5 min, using a planetary mixer (AR-100, THINKY Co. Ltd., Tokyo, Japan).

### 2.1. 3D Printing

Wood ink was extruded using a Hyrel3D 30 M (Hyrel International, Inc. Norcross, GA, USA) printer equipped with a disposable syringe extruder (SDS-10) with a 10 mL luer-lock syringe mounted with 1.626 mm (14 G) smoothflow tapered tip (Nordson EFD, USA). G-code files were prepared via Slic3r software, and objects were printed at 1 mm layer thickness with various speed rates as indicated in the text. Printing and drying of all samples were performed at room temperature.

Throughout the manuscript, we use the term ‘printing speed’ rather than ‘flow rate’ as it is more intuitive to understand and implement in other systems. The volumetric flow rate can be calculated by multiplying the cross-section (path width times layer thickness) with printing speed. For instance, a printing speed of $v=300\frac{\mathrm{mm}}{\min}$ corresponds to the volumetric flow of $Q=8\frac{{\mathrm{mm}}^{3}}{s}$. The 3D printing system allows for control of the flow rate using a stepper motor piston that dictates the exact amount of volume to be extruded at a given time.

### 2.2. Object Characterization

Warped objects dimensions were photographed using a 2D scanner (HP LaserJet 1536dnf MFP), and a digital camera (Sony a6000 equipped with Laowa 65 mm F2.8 lens). 16 specimens were measured for cylindrical samples and 18 specimens were measured for the helical samples.

### 2.3. Modeling and Simulation

Numerical optimization was performed in Python3, using the built-in optimizer of the SciPy package (scipy.optimize, v1.7.1) using the Limited-memory constrained Broyden–Fletcher–Goldfarb–Shanno algorithm (L-BFGS-B).

## 3. Results and Discussion

Wood warped objects were obtained by extrusion-based 3D printing technology using “wooden“ aqueous ink consisting of finely ground WF particles and plant-extracted natural binders from the plant cell wall, CNC and XG. Extrusion of such ink is possible due to CNCs rod-like particles that function as a rheological modifier, which result in a pseudoplastic rheological behavior. It means that at rest, the viscosity of the ink is high, and therefore prevents it from dripping from the extruder nozzle, but as pressure is applied by the syringe piston, the viscosity drops and enables the smooth flow of ink [19]. Once the liquid ink is deposited at its pre-designed location, the viscosity increases again and enables the fixation of the 3D object (extensive rheology measurements have been reported in our previous publication [2]). The applied pressure in extrusion-based printing systems has been shown to influence the alignment of the particles, which results in unique anisotropy of the printed objects [20,21].

Preliminary experiments showed that in our system, the anisotropic property resulted in a different shrinkage ratio of wet to dry objects, perpendicularly and parallel to the printing direction. Based on this understanding we printed wooden objects at different 2D predesign pathways and various speeds, while the obtained dried object was spontaneously warped into a 3D object.

**Figure 2:**
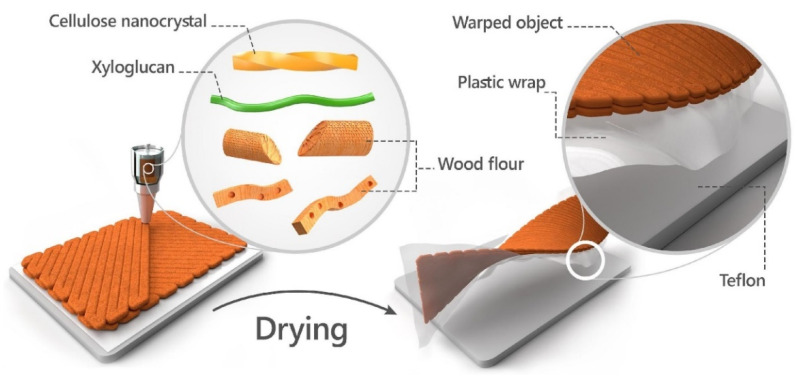
Schematic drawing of the printing and drying process. Wood ink consists of WF, and CNC and XG are extruded in a 2D predesign pathway on a plastic wrap placed on a Teflon substrate. The obtained object reduces its volume as a result of gradual evaporation of water and glides on the Teflon substrate. The anisotropic shrinkages result in a 3D wood warped object.

Due to the brittleness of dried wood and the printing process technique, obtaining an uncracked dried object is a major challenge. To reduce substrate influence on drying and the development of cracks, the printing process was conducted on a plastic, thin sheet placed on a Teflon substrate, as shown in the Figure 2 illustration. After an object was successfully printed, the plastic sheet was cut along the printed object, thus enabling the object and the plastic sheet to slide onto the Teflon substrate during drying. In addition, to reduce the non-homogeneous drying effect due to the contact with the substrate, we extended the drying process by keeping the object in a closed chamber, which prolonged the time to at least 48 h for complete drying. Typically, the mass decreased by 23.3% from printing to its final dry state.

To control the 4D behavior, we printed bilayer structures that differed in printing orientation and flow rate. When the specimens are dried, the difference in printing orientation results in a spontaneous bi-axial (double) curvature, that could vary in direction and magnitude [22]. The emergence of this curvature morphs the specimen’s shape. Finding the resulting shape involves an elastic calculation which is usually not tractable and can only be performed numerically [23]. The complexity stems from two competing tendencies of the sheet: the bi-axial spontaneous curvature favors configurations with saddle-like double curvature, while the flat lateral geometry of the sheet only admits a single curvature (cylinders and cones) without stretching. Clearly, the two tendencies cannot be simultaneously fulfilled in a single configuration. Therefore, the equilibrium configurations of such structures, known as incompatible sheets, are set by the competition between their stretching and bending energy terms. The former penalizes for non-vanishing Gaussian curvature and the latter for deviations of the curvature from the induced one [23,24]. In the thin limit, the sheet is unstretchable, obeying its in-plane geometry, hence having zero Gaussian curvature and the curvature being uniaxial everywhere (deviating from the induced one). This deviation costs bending energy. In the thick limit, the structure obeys the induced (double) curvature at the cost of stretching energy. This fact is the source for the shape transitions that appear in many such systems, where sheets that are made of the same material undergo shape transitions depending on their lateral geometry and thickness. Whenever the structure is elongated and is ribbon-like, such as the structures printed in this work, the shape transition also involves the width of the structure, where the wide and narrow ribbons correspond to the thin and thick limits, respectively.

**Figure 3:**
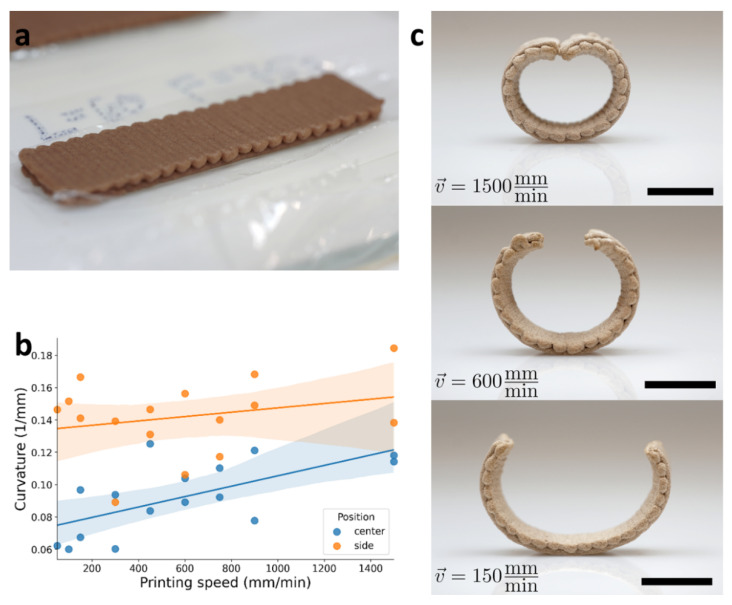
90° oriented bilayers. (**a**) printed object “as printed”, wet length = 60 mm, wet width = 15 mm. (**b**) Curvature as a function of printing speed measured at two different locations: center and side, blue and orange markers, respectively. (**c**) Dried 3D warped objects at different velocities, scale bar indicates 10 mm, demonstrating an increased effect at higher velocities.

To exploit the shape-programming capability of our system, we must first calibrate the induced curvature, $\kappa_{0}$. For that, we chose a configuration that would result in a robust shape that was easily measurable: rectangular objects consisting of two layers. The bottom layer was pre-designed so that the printing pathway lines moved parallel to the long axis of the rectangle, while the top layer was printed perpendicular to it (Figure 3a). Moreover, we hypothesized that different printing velocities (corresponding to different flow rates) would affect the microscopic anisotropy of the material that would be represented in the induced curvature. The structure of this rectangular object has an induced curvature of opposite signs, along with and across its long dimension. In either of the elastic limits, the solution is cylindrical, with a radius corresponding to the induced curvature. In the thin/wide limit, no perpendicular curvature should appear, and in the thick/narrow limit, the cylinder will have a saddle-like profile, similarly to a catenoid. In both cases, the radius of the cylinder corresponds to the magnitude of the induced curvature.

**Figure A1:**
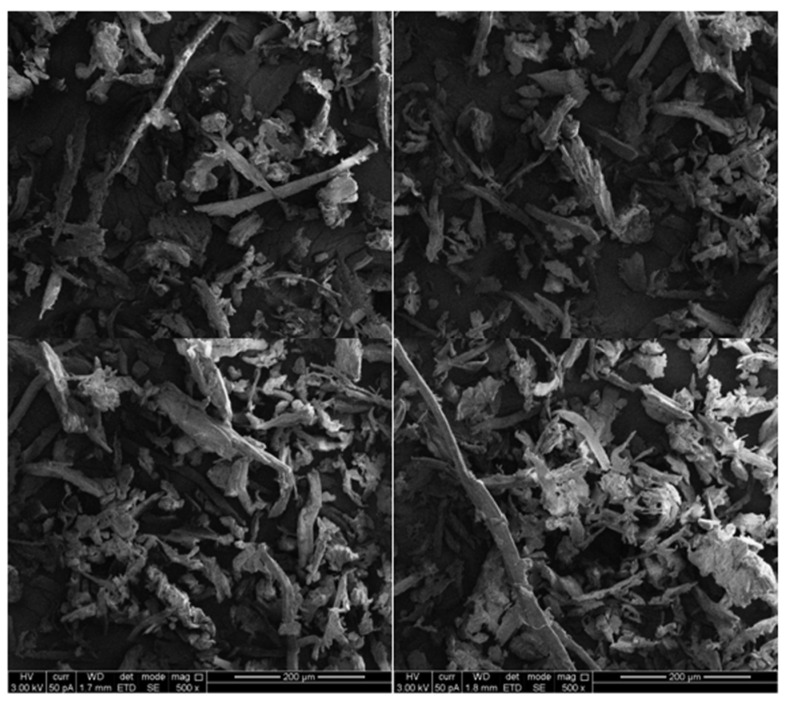
Wood flour particles in the powder starting material.

**Figure A2:**
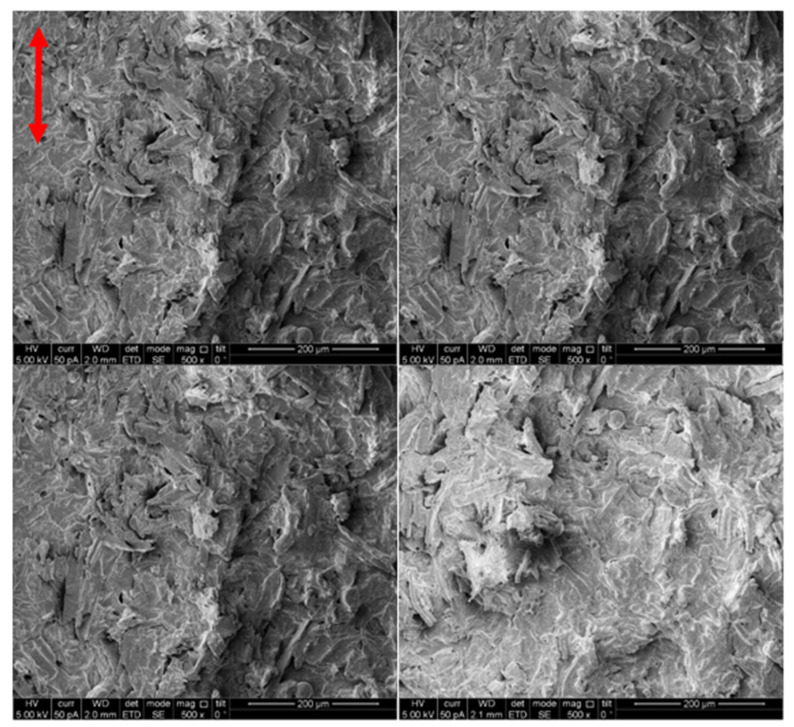
SEM images of a cylindrical sample printed at $\vec{v}=150\mathrm{mm}/\min$ (arrows indicating printing pathway direction.

**Figure A3:**
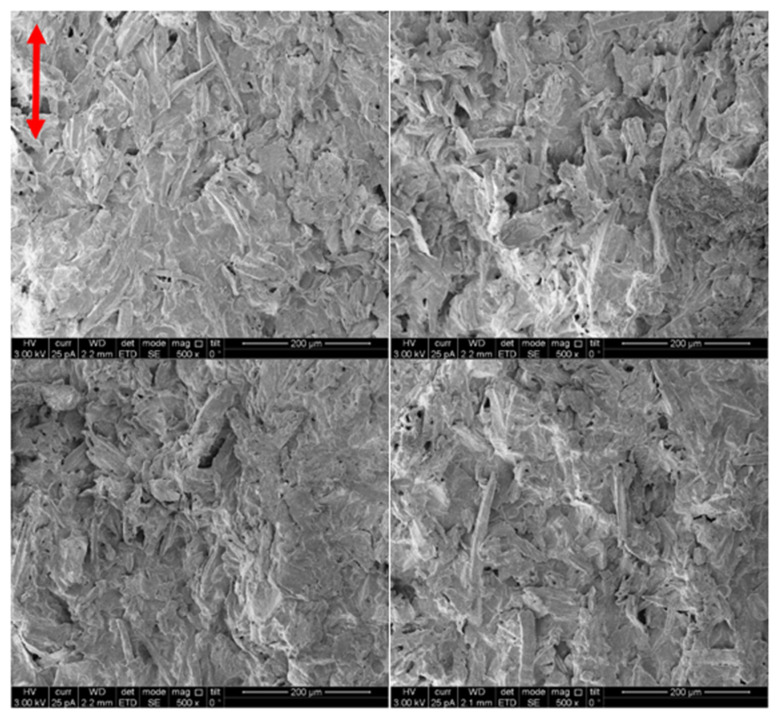
SEM images of a cylindrical sample printed at $\vec{v}=600\mathrm{mm}/\min$ (arrows indicating printing pathway direction.

**Figure A4:**
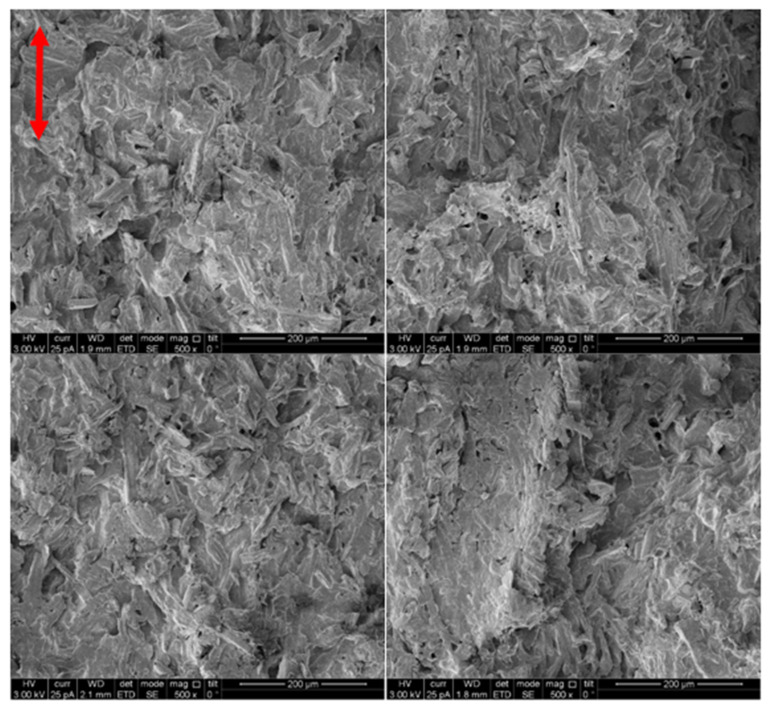
SEM images of a cylindrical sample printed at $\vec{v}=1500\mathrm{mm}/\min$ (arrows indicating printing pathway direction.

Next, we printed objects of varying lengths and varying printing speeds while maintaining the same object dimensions by adjusting the printing flow rate, which, upon drying, indeed adopted cylindrical shapes having varying radii. The dependence of the microstructure of the objects on the printing speed was evaluated by SEM imaging and is presented in Appendix A. The microstructures were evaluated after the objects were printed and wrapped. As-received wood particles are seen in sizes of tens of microns (Figure A1), in agreement with the materials data sheet (particle size of less than 75 µm size particle for 92% of particles and above 150 µm for 2% of the wood flour particles). At 150 mm/min, the ink seemed to have solidified into a uniform structure but with no preferred orientation (Figure A2). At higher printing speeds of 600 mm/min and 1500 mm/min, it appeared that some of the particles aligned with the printing pathway direction (Figure A3 and Figure A4 respectively). This finding, together with the macrostructure behavior of the wrapped object, suggests that particles do align with respect to the predesign pathway during the printing by extrusion.

**Figure 4:**
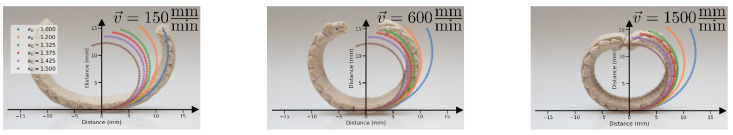
Applying the curvature model on 3D warped objects at different velocities. The dashed lines are the profiles resulting from our numerical model for different values of $\kappa_{0}$. The resulting values vary from 0.1 ${\mathrm{mm}}^{-1}$ to 0.14 ${\mathrm{mm}}^{-1}$.

For shape analysis, we took photos of these structures from a side-view and measured their circular profiles. However, our measurements revealed that in many cases, the radius of these profiles changes along with the ribbon, contradicting our prediction of cylindrical structures. Figure 3b shows the difference of the measured curvature at two places, the center and side of the ribbon. As can be seen, the measured curvature on the center of the ribbon was smaller than on the side. We hypothesize that this discrepancy is due to gravitational effects, resulting from the fact that the pre-design printing pathway dictated bending in a direction opposite to gravity. To test it, we made a simple model that minimizes the bending and gravitational energy of the profile (see Appendix B for more details). The model assumes a quasi-1D structure, with a spontaneous curvature ($\kappa_{0}$), bending rigidity (B), and gravitational energy density (ρgtz≡Gz), from which it can be deduced what is the final obtained curvature of the object (Figure 4). The behavior of this simple model looks almost identical to our experimental measurements and revealed some key insights regarding the system.

First, at the two edges of the ribbon, the curvature coincides with the induced one, and closer to its center the gravitational effects become more dominant. This was predicted, since a slight modification of the curvature near the center will reduce the height of the entire structure, thus significantly reducing the gravitation energy. In contrast, modifying the curvature at the edges will hardly reduce gravitation energy.

Moreover, the competition between gravity and bending energy introduces a new length scale, $\lambda \equiv \frac{B\kappa_{0}^{2}}{2G}$, which sets the typical height, ⟨z⟩, in which gravity becomes dominant. Whenever $\lambda^{-1}\langle z\rangle \gg 1$, gravity dominates the structure’s mechanics, and whenever $\lambda^{-1}\langle z\rangle \ll 1,$ gravity can be neglected.

Finally, if $\lambda^{-1}\langle z\rangle$ is close to one, when fixing the bending rigidity and varying the induced curvature only slightly, very large variations of the curvature are brought near the center (while the curvature near the edge follows the induced one and varies only a little). Therefore, we see again that the effect of gravity on the center of the ring is greater.

Based on this understanding, we measured the induced curvature, $\kappa_{0}$, by calculating the radius from the side of the ribbon, and the obtained curvature was found to be 0.135–0.145 1/mm, increasing with printing speed (Figure 3b). This observation adds another tier to the programmed wood warped geometry, by introducing a degree of alignment to the orientation of the material. Furthermore, we find that for λ≈21 mm, our model reproduces the large variation in the curvature at the center of the ribbon. For the formed ribbons, L≈60 mm which corresponds to a configuration with a typical height of about 10 mm, hence $\lambda^{-1}\langle z\rangle \approx 0.5$, and therefore it fits an intermediate regime in which the shape is dominated by bending but gravitational effects cannot be neglected.

**Figure 5:**
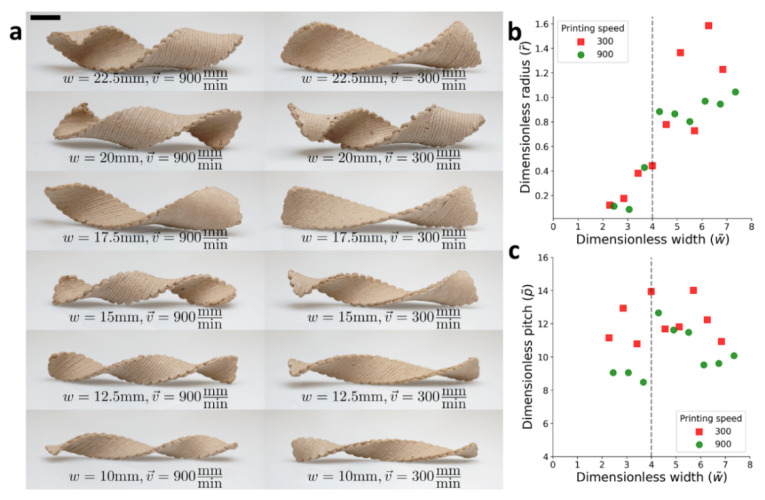
45° oriented bilayers. (**a**) Dried, 3D-warped helical objects at different velocities and width, scale bar indicates 10 mm. (**b**) Dimensionless radius versus dimensionless width. (**c**) Dimensionless pitch with versus dimensionless width. The vertical dotted line represents the threshold of the shape transition.

Following the cylindrical structure calibration, helical architecture was accomplished by predesigning printing pathways to be not only orthogonally between the two layers but oriented with an angle of $\pm 45^{{}^{\circ}}$ to the rectangular, long axis (Figure 5). Such an architecture appears in seedpods and self-assembled macromolecules, and is known to generate helical structures [25]. As opposed to the cylindrical orientation, now the shape transitions were characterized by the radius and pitch of the warped structure. Narrow wooden bilayers objects obey the (saddle-like) reference curvature and therefore warped into a twisted structure with a vanishing radius. At the other limit, to avoid stretching, wide objects are warped into helical structures cut from a cylinder, which follows one of the prescribed principal curvatures as much as possible, while developing no Gaussian curvature.

Objects of varying widths (10–30 mm) were printed, at the two velocities, 300 and 900 mm/min. Again, the wet samples were rectangular (L= 900 mm) and flat, but upon drying, they adopted striking helical configurations, which converted from twisted to helical configurations (Figure 5a). Each warped object was photographed, and the radius and pitch were measured. As expected, samples with the same lateral dimensions printed at different velocities adopted different configurations. In the wide regime (W), the radius of the helical structures should be $r_{W}\approx 1/\kappa_{0}$, which corresponds to curvatures of 0.14 and 0.1 ${\mathrm{mm}}^{-1}$ for the higher and lower velocities, respectively (Figure 5b). These values are similar to what was found in the calibration experiments. The pitch in this limit is given by $p_{W}\approx 2\pi /\kappa_{0}$, which for our calibrated values should read about 50 and 40 mm for the higher and lower velocities, respectively (Figure 5c). The values we measured were somewhat smaller. Previous studies found the transition to be around $w_{c}\approx 4\sqrt{\frac{t}{\kappa_{0}}}\approx 15\;\mathrm{mm}$ which is where the samples convert from helical into twisted structures [24]. Near the transition, the radius decreases rapidly, and the pitch spikes. Finally, at the narrow regime (N), the radius vanishes as expected, and the predicted pitch again reads $p_{N}\approx 2\pi /\kappa_{0}$.

Overall, the experimental results qualitatively matched the predicted behavior. Furthermore, we found good quantitative agreement for the critical width of the transition and the behavior of the sample printed at high velocity. As argued above, the samples printed at a lower velocity seemed to be more sensitive to gravity, which can prevent them from twisting into the right shape throughout the drying process.

**Figure 6:**
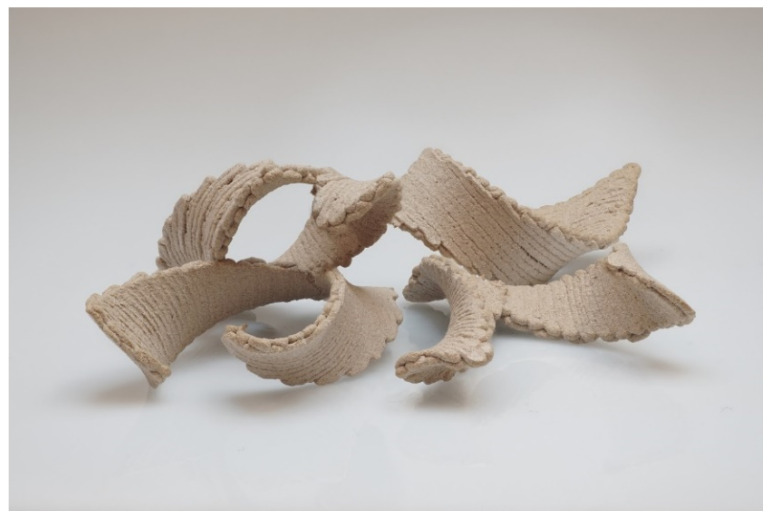
3D printed wood warped seedpod. These were made by printing to helical ribbons of opposite chirality with a relative angle of 45°.

Once we have established the possibility to control the shape up to helical structures, by tailoring the orientation and alignment, this two-knob simple concept, can serve as a new toolkit to create wooden objects with many complex structures. By introducing different combinations of the two knobs, we could create 3D printed seedpod-like structures, as shown in Figure 6, which is an excellent starting point for the fabrication of more complicated objects.

## 4. Conclusions

In this work, we presented how using wooden ink with a raw industrial polydisperse wood powder with a carefully controlled 3D printing system can result in morphing wood objects. We printed elongated ribbons composed of two perpendicular layers from two families of structures: cylinders, in which the printing direction matched the axes of the ribbon, and helices, in which the printing direction was rotated by ±45° with respect to these axes. The cylindrical structures were used to estimate the induced curvature and to gauge the magnitude of gravitational effects on the shape. The helices displayed a shape transition from a twisted structure to a flat helix. It was found that the critical width, associated with this transition, agrees with the theoretical prediction.

Different from previous reports, we not only predesigned the 3D printing pathway, but we controlled the ink flow rate, thus enabling orientation and degree of alignment. These results validate our hypothesis that CNC particles affect the shear alignment induced by the extruding process. Here, for simplicity, we printed only along straight lines. but introducing curved lines [26], zigzags [27], and fractal Hilbert patterns [28] will greatly extend the possible outcomes. Furthermore, the presented approach can be implemented in other systems, such as hydrogels, and by carefully altering the characteristics of the ink, the effect of printing velocity on the induced orientation can be enhanced.

In conclusion, 3D printed wooden structures were successfully designed and fabricated to evolve through time into programmed warped geometry, by controlling both the direction via the printing predesigned pathway and the anisotropy by adjusting the printing flow rate. We have found that Gaussian curvature can be induced for continuous wooden surfaces, thus enabling fully biomimic control of objects, by tailoring both materials’ composition and the drying processes of wood. This opens new possibilities toward the 4D printing of wood.

## Data Availability

The data presented in this study are openly available in the form of a Jupyter notebook in FigShare at doi:10.6084/m9.figshare.17311544.

## Appendix A. Microstructure of the Warped Objects

Samples microstructure were evaluated after objects were printed and let for warping using SEM imaging (XHR Magellan 400L).

## Appendix B. Gravitational Effects

To assess the magnitude of the gravitational effects we used a reduced 1D model of the problem: an Elastica of length ℓ and thickness t is subject to a spontaneous curvature $\kappa_{0}$ and gravity. Our approach was to find the configuration that minimizes the total energy $E=E_{B}+E_{g}$.

We use the arclength coordinate u to express both energy densities ${ℇ}_{B}$ and ${ℇ}_{g}$, satisfying $E=\int^{\;}\left({ℇ}_{B}+{ℇ}_{g}\right)du$. The gravitational energy density is simply

$${ℇ}_{g}=t\rho gz\left(u\right)\equiv Gz\left(u\right)$$

where g is the gravitational acceleration, ρ is the mass density of the material, and z(u) is the local height of the configuration. The bending energy is given by

$${ℇ}_{B}=\frac{1}{2}B{\left(\kappa \left(u\right)-\kappa_{0}\right)}^{2}$$

where B is the bending rigidity of the material, that for an isotropic plate is given by

$$B=\frac{t^{3}Y}{12\left(1-\nu^{2}\right)}$$

where Y and ν are the Young modulus and Poisson ratio of the material, respectively.

For simplicity, by assuming that both halves of the ribbon are symmetric and non-dimensionalizing both energy terms, we minimize the reduced energy

Eeff=∫0ℓ/2((κ(u)κ0−1)2+λ−1z(u))du

where, $\lambda =\frac{B\kappa_{0}^{2}}{2G}=\frac{t^{2}Y\kappa_{0}^{2}}{24\rho g\left(1-\nu^{2}\right)}$, is a length scale setting the ratio between the gravity and bending energy magnitudes compared to the typical height, ⟨z⟩ (i.e., $\lambda^{-1}\langle z\rangle \gg 1$ corresponds to situations where gravity dominates the structure’s mechanics).

Finally, the two energies depend on different yet dependent variables, hence we aim to express the z coordinate using the curvature κ.

Note that from the definition of the curvature, dθ=κ(u)du, and $dz=\kappa^{-1}\sin\theta d\theta =\sin\theta \;du$ (and naturally, dx=cosθdu). Then by assuming $z\left(u=0\right)=\partial_{u}z\left(u=0\right)=0$ (the center is at height 0 and is horizontal)

$$\theta \left(u\right)=\overset{u}{\underset{0}{\int }}\kappa \left(u^{\prime }\right)du^{\prime }$$

and finally, the configuration is given by

$$z\left(u\right)=\overset{u}{\underset{0}{\int }}\sin\left[\overset{u^{\prime }}{\underset{0}{\int }}\kappa \left(u^{\prime \prime }\right)du^{\prime \prime }\right]du^{\prime },x\left(u\right)=\overset{u}{\underset{0}{\int }}\cos\left[\overset{u^{\prime }}{\underset{0}{\int }}\kappa \left(u^{\prime \prime }\right)du^{\prime \prime }\right]du^{\prime }$$

For the numerics, we discretize the ribbon into N identical segments of length δ:

Eeff=∑j=1N[δ(κjκ0−1)2+λ−1δ2∑k=1jsin(∑m=1kδκm)]

Estimating the bending rigidity and Young modulus:

We find

$$\frac{B\kappa_{0}^{2}}{2t\rho g}\equiv \lambda =21\;\mathrm{mm}$$

Assuming $\rho =1.4\times 10^{3}\;\mathrm{kg}/m^{3}$ and $g=9.8\;m/s^{2}$ and taking t=80% of 2 mm=1.6 mm and $\kappa_{0}=0.14\;{\mathrm{mm}}^{-1}$ we can find the bending rigidity

$$B=\frac{2t\rho g\lambda }{\kappa_{0}^{2}}=4.7\times 10^{-5}\;J$$

Now, assuming an isotropic material the Young modulus is:

$$Y=\frac{12\left(1-\nu^{2}\right)B}{t^{3}}=120\;\mathrm{kPa}$$
